# Developing Bovine Brain-Derived Extracellular Matrix Hydrogels: a Screen of Decellularization Methods for Their Impact on Biochemical and Mechanical Properties

**DOI:** 10.1021/acsomega.3c04064

**Published:** 2023-09-29

**Authors:** Duygu Turan Sorhun, Alican Kuşoğlu, Ece Öztürk

**Affiliations:** † Engineered Cancer and Organ Models Laboratory, Koç University, Istanbul 34450, Turkey; ‡ Research Center for Translational Medicine (KUTTAM), Koç University, Istanbul 34450, Turkey; § Department of Medical Biology, School of Medicine, Koç University, Istanbul 34450, Turkey

## Abstract

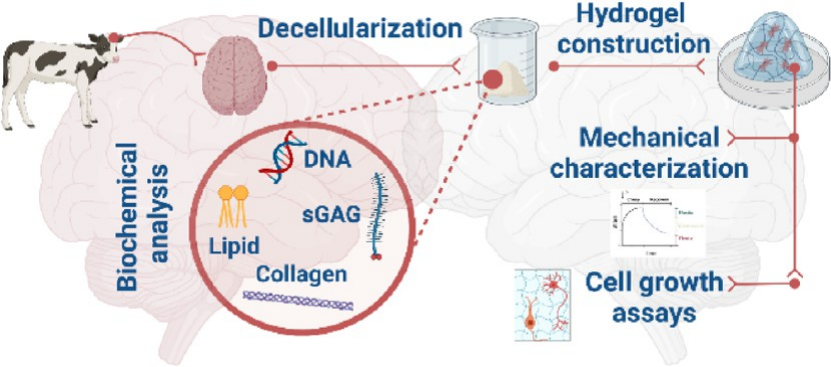

Tissue models that recapitulate the key biochemical and physical aspects of the brain have been highly pursued in neural tissue engineering. Decellularization of native organs offers the advantage of preserving the composition of native extracellular matrix (ECM). Brain ECM has distinct features which play a major role in neural cell behavior. Cell instructive ligands and mechanical properties take part in the regulation of cellular processes in homeostasis and diseases. One of the main challenges in decellularization is maintaining mechanical integrity in reconstituted hydrogels and achieving physiologically relevant stiffness. The effect of the decellularization process on different mechanical aspects, particularly the viscoelasticity of brain-derived hydrogels, has not been addressed. In this study, we developed bovine brain-derived hydrogels for the first time. We pursued seven protocols for decellularization and screened their effect on biochemical content, hydrogel formation, and mechanical characteristics. We show that bovine brain offers an easily accessible alternative for *in vitro* brain tissue modeling. Our data demonstrate that the choice of decellularization method strongly alters gelation as well as the stiffness and viscoelasticity of the resulting hydrogels. Lastly, we investigated the cytocompatibility of brain ECM hydrogels and the effect of modulated mechanical properties on the growth and morphological features of neuroblastoma cells.

## 1 Introduction

The extracellular matrix (ECM) is the fundamental noncellular unit in native tissues whose main components include macromolecules such as proteoglycans (PGs) and fibrous proteins, such as collagen, elastin, laminin, fibronectin, and water.^1^ The ECM provides physical scaffolding, sets the ground for intercellular interactions with cell instructive constituents, and contributes to regulation of biological functions involved in homeostasis, migration, differentiation, and pathological progress. During development, tissues attain a unique microenvironmental composition and topology within the ECM. In addition to the differences in tissue types, alterations such as aging, wounding, or disease progression lead to dynamic changes in the ECM content and organization.^2,3^

The use of three-dimensional (3D) human tissue models in neuroscience gained attention over conventional two-dimensional (2D) cultures as 2D culture models are insufficient in displaying the key aspects of the brain microenvironment in terms of supporting cellular processes such as synaptogenesis, differentiation, neurodevelopment, neuronal maturation, and cell–cell signaling.^3,4^ To mimic native or disease microenvironments in 3D tissue models, the use of natural or synthetic polymers has been widely pursued.^5^ Decellularization of tissues through removal of cellular and nuclear components and its reconstitution into scaffolds have been a promising approach to generate 3D tissue models with the ability to recapitulate native microenvironments.^6^ The decellularization process requires methodical optimization according to the composition and properties of the tissue of interest. By a broad classification, physical, chemical, and biological decellularization methods can be performed, and each treatment has a different impact on the resulting composition of decellularized tissue.^7^ Physical methods are often preferred in conjunction with chemical and enzymatic treatments to improve the penetration rate of solutions.^8,9^ Freeze–thaw, a frequently pursued approach for physical disruption of cellular material via formation of ice crystals in tissues, also exerts detrimental effects on the ECM.^9^ Homogenization is mostly applied to eliminate lipid content during decellularization of lipid-rich tissues such as the pancreas or adipose tissue.^10^ Chemical treatments comprise nonionic detergents such as Triton X-100, ionic detergents such as sodium dodecyl sulfate (SDS) and sodium deoxycholate (SDC), and acids, including peracetic acid. SDS stands out as a preferred choice of detergent in the literature due to its harmless effect on collagen and elastin.^9^ Ensuring the appropriate concentration and treatment duration of SDS is crucial to avoid protein denaturation in the ECM.^11^ On the other hand, SDC exhibits beneficial effects on decellularization; however, it has a more disruptive impact on the native tissue infrastructure.^7^ Due to its nonionic nature, Triton X-100 is expected to have the least damaging impact on protein structures, and it was shown that applications with Triton X-100 resulted in varying output and efficiency depending on the applied tissue.^7,12^ Peracetic acid has been shown to effectively disrupt cell membranes with no adverse effects on ECM proteins and biomechanical properties of decellularized tissue.^7^ Ethanol and dichloromethane are frequently used reagents for decellularizing spinal cord and sciatic nerve tissues, aimed at effective removal of lipids.^13^ To facilitate the fragmentation of residual deoxyribonucleic acid (DNA), various biological agents, including trypsin, and endonucleases such as deoxyribonuclease (DNase) can be employed.^8,14^

During the engineering of biological scaffolds, it is important to formulate decellularization strategies according to the distinct properties of the target tissue. The brain, a uniquely delicate tissue, is distinguished from other organs by its outstanding features, such as limited stromal space with well-defined ultrastructure and low amount of fibrous ECM proteins, including fibronectin, vitronectin, and collagen, and high glycosaminoglycan (GAG)-to-collagen ratio.^15−18^ Furthermore, brain tissue is highly enriched in lipid content; however, integration of delipidization strategies into the brain decellularization process has not often been adopted in the literature.^10^ Eventually, the ultimate goal for an optimized decellularization methodology is efficient cell removal with maximum preservation of native ECM.^19^

Although decellularized tissue-derived ECM (d-ECM) hydrogels offer conservation of biochemical complexity of native tissues, one main challenge in their use is to maintain mechanical stability.^20,21^ Understanding the effect of decellularization methods on the biochemical content of decellularized tissues and the subsequent mechanical properties of reconstituted hydrogels such as stiffness and viscoelasticity is crucial to conduct studies within mechanically controlled microenvironments.^22^ The brain, possessing a very soft structure, is confined within the skull, which acts as a hard boundary for protection.^23^ Rodent and human brain tissue exhibit viscoelastic behavior with a Young’s modulus alternating between 0.1 and 16 kPa.^24^ Neuronal cells prefer soft matrices such as ∼700 Pa for growth and maturation, but too soft materials have also been shown to hamper neurite extension.^5,23,25^ The importance of mechanotransduction, conversion of mechanical stimulus into downstream signaling pathways, and its role in the regulation of brain homeostasis have been emphasized in the field.^23^ Studies have demonstrated that changes in brain elasticity and viscoelasticity occur in both aging and disease conditions.^26−28^ For instance, it has been shown that there is reduced stiffness and viscosity in the hippocampus of Alzheimer’s disease patients.^29^ Moreover, reduction of viscoelasticity in the brains of patients with multiple sclerosis was observed.^26,30^ The implications of such findings for the ECM-related biomechanical changes reveal the need for mechanically controllable scaffolds for disease modeling efforts in neuroscience. The effect of decellularization on the brain-derived hydrogel viscoelasticity has not been previously addressed. Therefore, an elaborative mechanical characterization of decellularized brain tissue-derived ECM (db-ECM) hydrogels presents an essential need in the field.

Several studies have demonstrated successful decellularization of brain tissue from various species, including porcine and humans.^15,31−33^ In DeQuach et al.’s study, porcine brain tissue was decellularized using SDS, yielding a collagen, laminin, and perlecan-rich matrix and was used as a coating material for human-induced pluripotent stem cell-derived neuron culture.^31^ They also showed the injectability and *in vivo* gelation capability of the db-ECM hydrogels. Sood et al. developed another model to show the support of fetal porcine brain ECM on neural growth and differentiation. Their model involved a donut-shaped structure to mimic the white matter at the center and the gray matter in the surroundings. They also performed decellularization of adult and fetal porcine brains and investigated the impact of ECM from different stages on neuronal growth.^15^ In a further study done by Jung et al., composite brain hydrogels with anisotropic orientation were constructed using decellularized porcine brain tissue. They argued that this method allowed for a better mimicking of brain tissue anatomically, and consequently, neuronal network formation was enhanced with augmented dynamic signaling.^33^ Koh et al. established human, patient-derived decellularized brain tissue for recapitulation of tumor microenvironment in glioblastoma. They argued that the use of organ-specific decellularized tissues might provide a more physiologically relevant matrix in tumor models.^32^

Bovine (*Bos taurus*) is a promising source for brain ECM studies in the neuroscience field as bovine brain has high similarity to human brain, with its large size and gyrencephalic structure, which is the state of folding and convolutions within the cerebral cortex.^34,35^ Another advantage of using bovine tissues in 3D culture experiments is their easy accessibility from local slaughterhouses.^34^ Recently, various bovine tissues, such as ovarian tissue,^36^ flexor tendons,^37^ retina,^38^ spinal cord meninges,^39^ endometrium,^40^ vocal fold^41^ and lung,^42^ were used as a source in decellularization procedures to fabricate hydrogels. Beachley et al. demonstrated fabrication of d-ECM nanoparticles from the white and gray matter of bovine brain, which were incorporated into hyaluronic acid–based hydrogels to develop an anatomically inspired core–shell spinal cord model in which neuronal differentiation was investigated.^43^ Development of reconstituted d-ECM hydrogels derived from bovine brain has not yet been pursued.

In this work, we fabricated reconstituted hydrogels from decellularized bovine brains for use in brain tissue engineering with a faithful recapitulation of the native tissue microenvironment. We pursued seven different protocols and thoroughly characterized their effect on the removal of cellular content, biochemical composition, and gelation capability and kinetics of reconstituted d-ECM and mechanical properties of d-ECM hydrogels, including stiffness and viscoelasticity, *in vitro* cytocompatibility, cellular growth, and morphology. Our data reveal that the choice of decellularization method highly impacts the gelation kinetics and mechanical stability of db-ECM hydrogels. Furthermore, the decellularization strategy changes the elastic and stress-relaxing behavior of hydrogels, which allows mechanical tunability and independent investigation of these characteristics for their effect on cellular morphology and behavior. Overall, bovine brain-derived d-ECM hydrogels provide a physiologically relevant, cytocompatible microenvironment that offers promising opportunities for *in vitro* modeling of neurodegenerative diseases and brain tumors.

## 2 Results

### 2.1 Fabrication of Decellularized Brain Tissues and Confirmation of Nuclear Content Elimination

**Figure 1:**
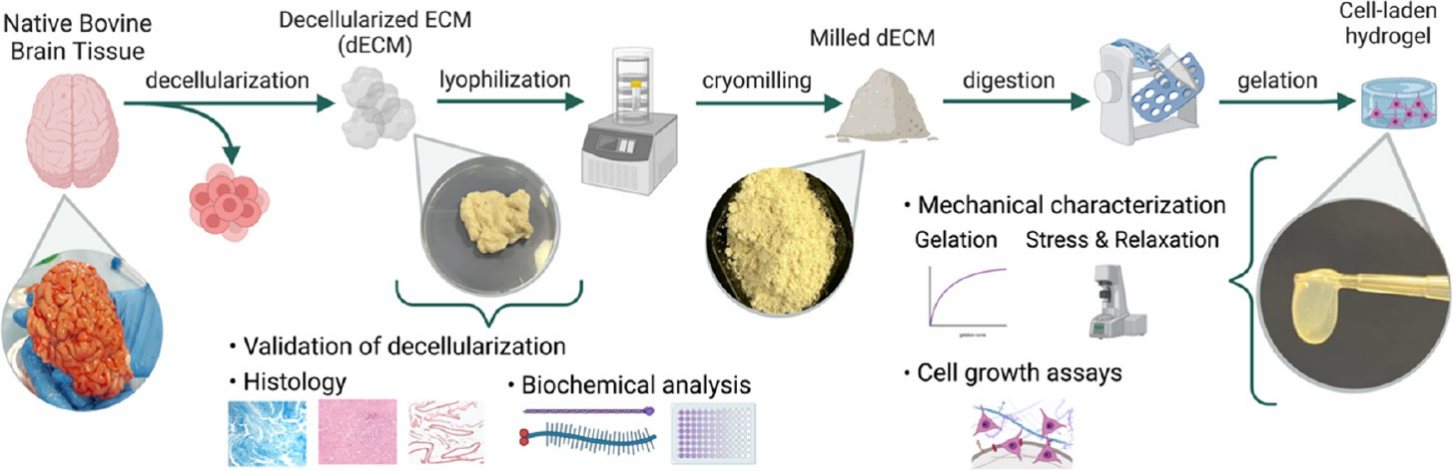
Pipeline for generation of db-ECM hydrogels from native bovine brain.

**Figure 2:**
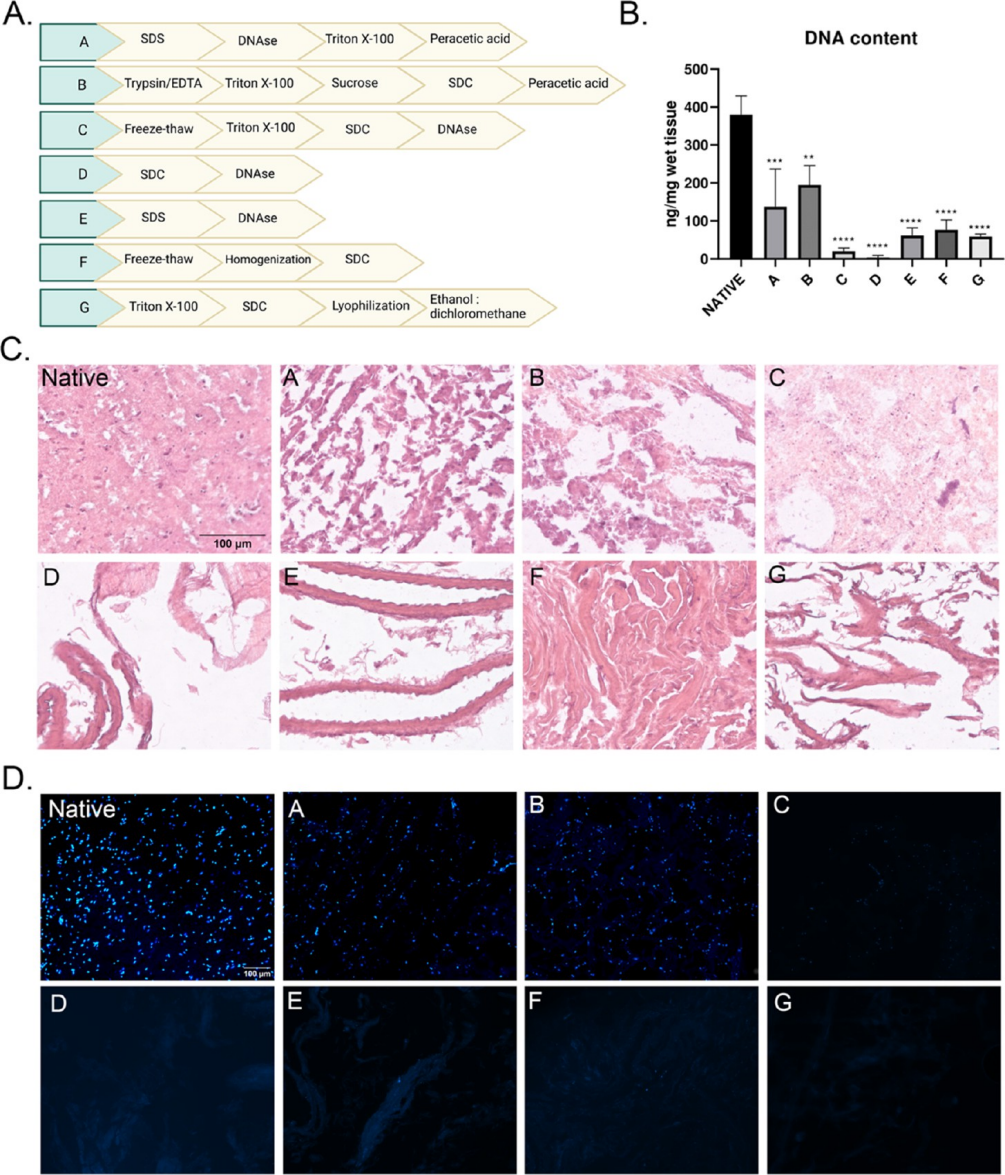
Decellularization methods applied and confirmation of decellularization. (a) Scope of different decellularization methods, (b) quantification of double-stranded DNA content, (c) histological examination with H&E staining (scale bar: 100 μm), and (d) Hoechst staining of native and decellularized tissues (scale bar: 100 μm).

The pipeline for fabrication of db-ECM hydrogels involved decellularization, lyophilization, cryomilling, and enzymatic digestion, bringing the digest ECM to physiological pH and thermal gelation, as summarized in Figure 1. Seven different decellularization protocols were employed and evaluated for their impact on efficient elimination of nuclear content and retention of native ECM constituents such as collagen and sulfated GAGs (sGAGs), gelation capability and kinetics, and mechanical properties of reconstituted hydrogels and cytocompatibility. Physical, chemical, biological, or certain combinations of these methods were applied for decellularization of bovine brain tissue (Figure 2a).

Following the different decellularization protocols, elimination of the cellular content was assessed in db-ECM samples (Figure 2b,d). First, the efficiency of the decellularization processes was confirmed by the loss of DNA content, which was assessed with the PicoGreen assay (Figure 2b). DNA content was significantly reduced in each decellularized sample when compared with the native bovine brain tissue, whereas methods C, D, E, and G appeared to perform with more efficient DNA removal. Residual DNA was measured as acceptably low as 20.25 ± 9 ng per mg wet tissue for option C, 3.45 ± 5 ng DNA per mg wet tissue for option D, 62.01 ± 20 ng/mg wet tissue for option E, and 59.16 ng/mg wet tissue for option G when compared to that of native tissue, which was 380 ± 49 ng DNA/wet tissue. These results were also confirmed qualitatively with H&E, Hoechst staining, and gel electrophoresis (Figure 2c,d and Supporting Information Figure 1). Hoechst staining clearly indicated remnants of nuclear content with methods A and B, whereas other options were successful at DNA elimination (Figure 2d). Decellularized tissues also demonstrated structural differences with varying methods. Methods D, E, and G revealed a distinct compactization of ECM when compared to native tissue and other decellularization methods (Figure 2c).

### 2.2 Biochemical Characterization of Decellularized Tissues

**Figure 3:**
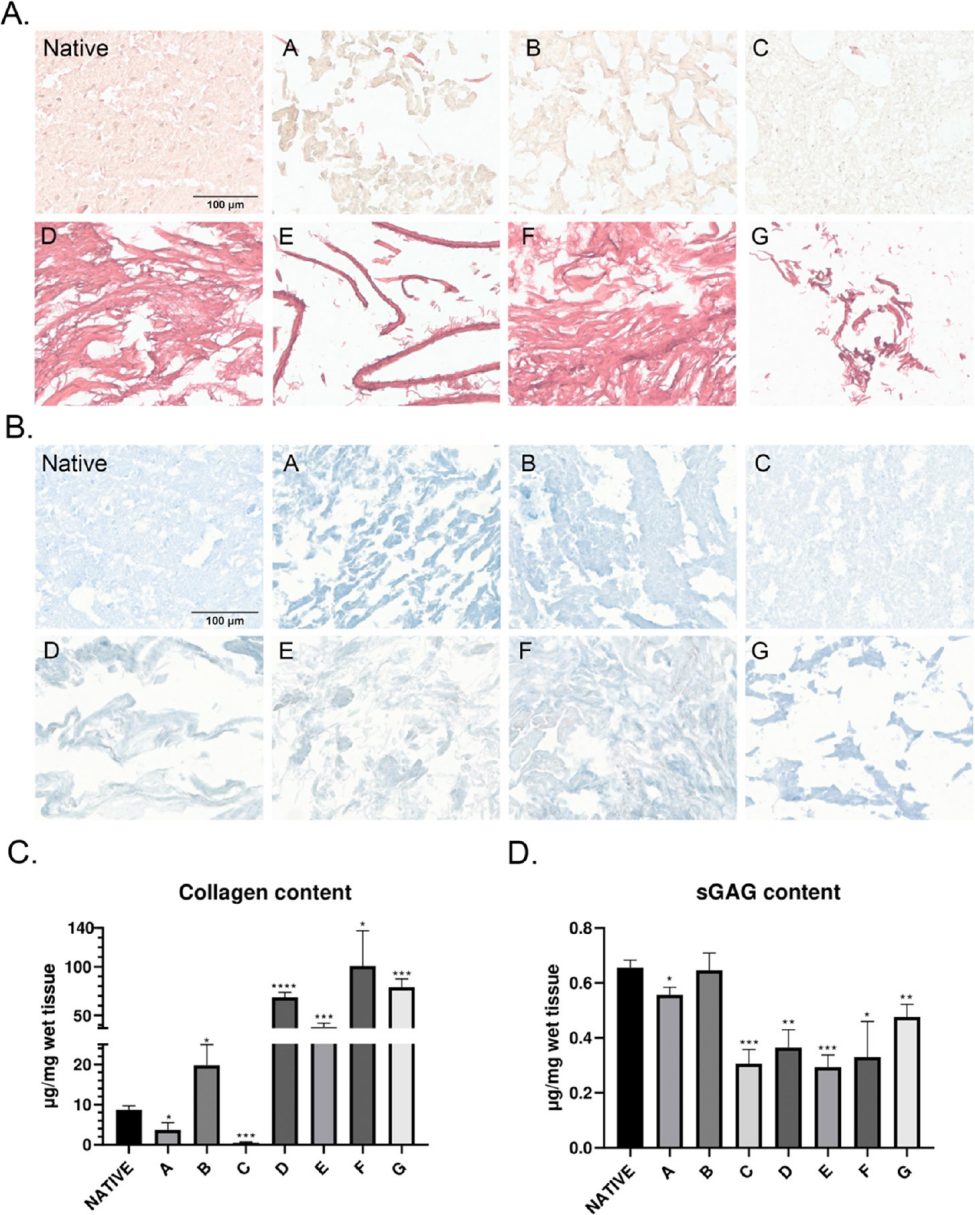
Evaluation of the ECM protein retention in decellularized samples. (a) Assessment of collagen content by Sirius red staining, (b) assessment of sGAG content by Alcian blue staining (scale bar: 100m), (c) quantification of insoluble collagen content, and (d) quantification of sGAG content.

After decellularization was confirmed by showing nuclear content elimination, the biochemical composition of the decellularized tissues was analyzed. As shown in Figure 3, collagen and sGAG were both quantitatively and qualitatively examined. An extreme increase in the insoluble collagen content was observed in options D (69 μg/mg), E (38 μg/mg), F (101 μg/mg), and G (79 μg/mg) in comparison to the native bovine brain tissue sample (9 μg/mg) which was also confirmed with Sirius red staining, as dense collagen fibers were explicitly shown (Figure 3a,c). This was due to extensive volume shrinkage during the decellularization process, upon which collagen became significantly more concentrated per weight of decellularized tissue compared to native bovine brain tissue. Especially, methods E and G gave rise to compact collagen fibril constructs, whereas in methods D and F, dispersed fibrils were present. On the other hand, lower collagen content was obtained in the samples A (4 μg/mg) and C (1 μg/mg). Furthermore, according to the quantitative assay based on 1,9-dimethylmethylene blue dye, it was concluded that sGAG levels were retained in options A and B and partially in option G. Treatments in other decellularization methods (C, D, E, and F) caused a decrease in sGAG content in decellularized tissues (Figure 3d). This decrease in sGAG content in the samples with C, D, E, and F decellularization options was also validated with Alcian blue staining (Figure 3b). Briefly, different decellularization agents had varying effects on the preservation of ECM components. Oil Red O staining was performed to assess the lipid content in native and decellularized brain tissues (Supporting Information Figure 2). Interestingly, methods D and E yielded the most effective lipid removal, even more so compared to methods F and G, which incorporated specific steps for delipidization.

### 2.3 Gelation of Reconstituted Bovine db-ECM Hydrogels

**Figure 4:**
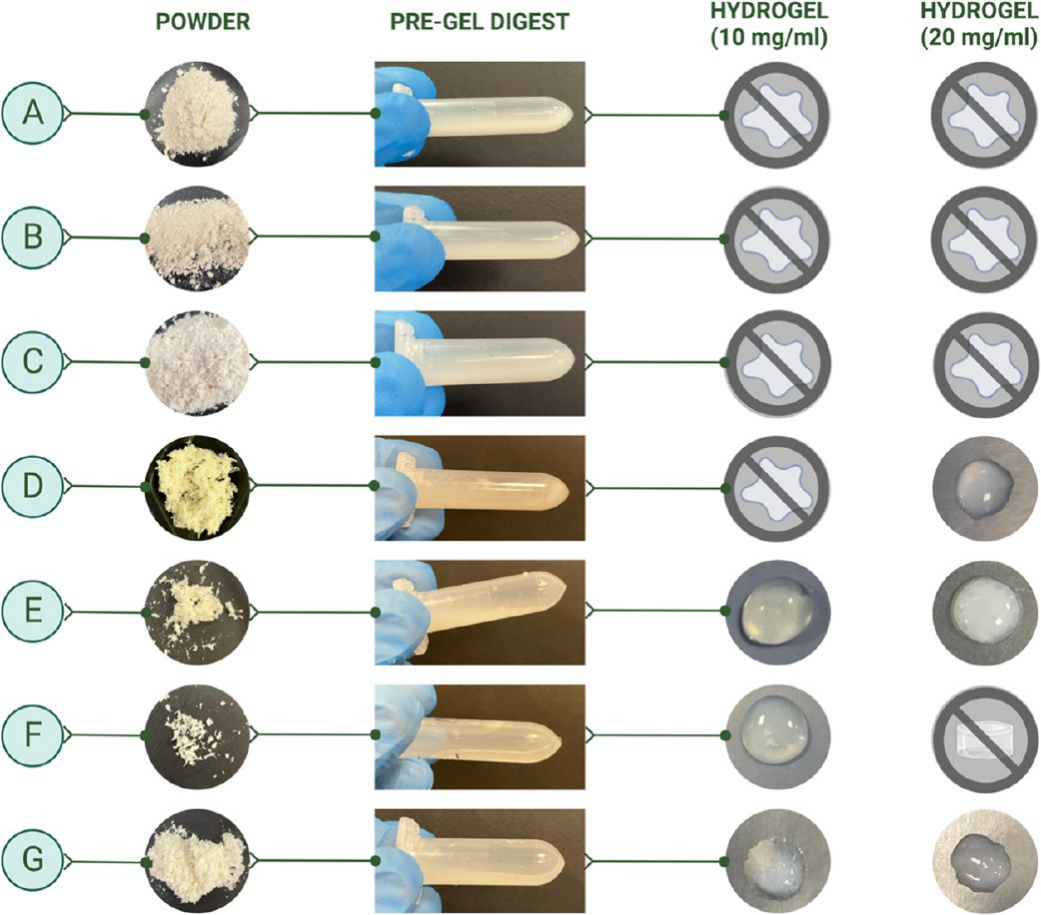
Representation of db-ECM solubilization and gelation: (left panel) db-ECM powder samples from different methods (A–G) obtained after cryomilling; (middle panel) pregel digest samples prior to neutralization and gelation; and (right panel) db-ECM hydrogel formation through thermal cross-linking at 37 °C from digests with either 10 or 20 mg/mL db-ECM concentration.

ECM samples derived from decellularized bovine brain tissues via the methods described above were used to fabricate reconstituted hydrogels. Among these, only db-ECM obtained with methods D, E, F, and G demonstrated successful gelation and hydrogel formation (Figure 4). In Figure 4, “pregel digest” images represent acidic liquid forms of digests for 10 mg/mL concentrations after 24 h of pepsin digestion, indicating efficient solubilization of db-ECM with no visible immature gelation. Horizontally placed tubes reveal the flow of digested ECM in liquid form. After incubating the neutralized digests at 37 °C for thermal gelation, options D–G showed intact gel forms consistently, whereas A–C failed (Figure 4). The effect of d-ECM concentration in the digest on gelation was also assessed. For method D, a db-ECM concentration of 20 mg/mL was optimal to ensure gelation, whereas, for method F, 10 mg/mL yielded the most homogeneous and efficient gel formation. Method F d-ECM at 20 mg/mL exhibited premature gel formation after digestion prior to neutralization and thermal gelation. On the other hand, methods E and G exhibited a wider range for optimal gelation, and both concentrations resulted in intact and homogeneous gel formation. Hydrogels with higher concentration of db-ECM demonstrated increased opacity and toughness in handling. Overall, these methods allowed for tunability of db-ECM content, hence ligand density, while ensuring successful gelation.

### 2.4 Mechanical Characterization of db-ECM Hydrogels

**Figure 5:**
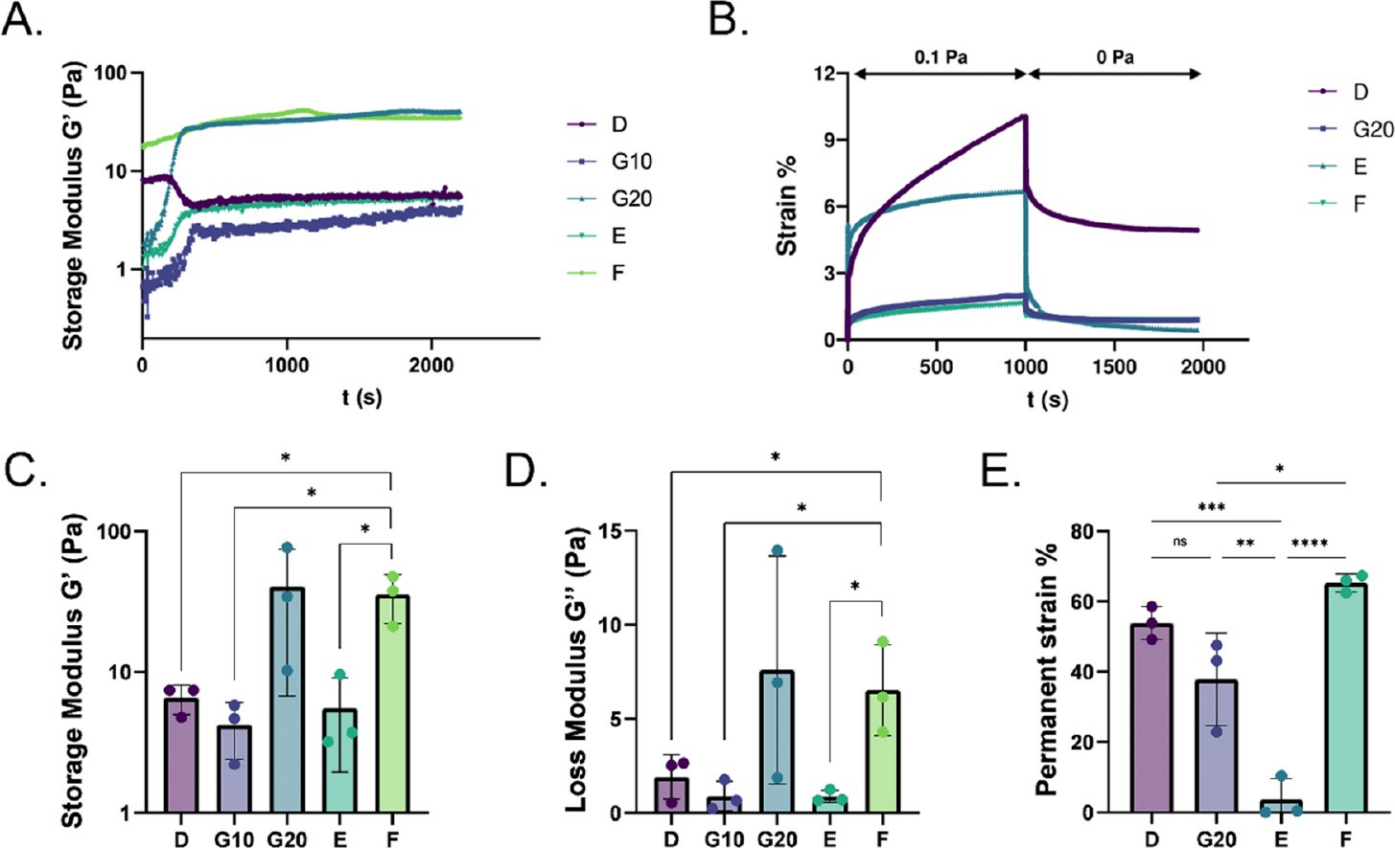
Assessment of gelation and mechanical properties of the db-ECM hydrogels. (a) Storage modulus of hydrogels (D, E, F, and G10 indicate hydrogels with 10 mg/mL db-ECM concentration; G20 hydrogels indicate 20 mg/mL db-ECM concentration). (b) Creep-recovery analyses of hydrogels. (c) End point storage modulus of db-ECM hydrogels after time-sweep. (d) End point loss modulus of db-ECM hydrogels. (e) Permanent strain % of db-ECM hydrogels obtained from creep-recovery test.

Mechanical properties of the db-ECM hydrogels were assessed by oscillatory rheology. Pregels obtained by decellularization methods D (SDC-based), E (SDS-based), F (physical-SDC-based), and G (Triton X-100-SDC-delipidization-based) were able to form hydrogels in 10 min during the temperature increase up to 37 °C and exhibited complete gelation in all trials (Figure 5a). Furthermore, the storage moduli of all hydrogel samples were higher than their loss moduli, confirming the gelation capability of the db-ECM hydrogels (Figure 5c,d). At a db-ECM concentration of 10 mg/mL, method F (physical-SDC-based) yielded hydrogels with higher stiffness compared to methods D (SDC-based), G (Triton X-100-SDC-delipidization-based), and E (SDS-based), which yielded similar storage moduli. Using method G (Triton X-100-SDC-delipidization-based), hydrogels with a 20 mg/mL db-ECM concentration had a stiffness range that was comparable to hydrogels of method F (physical-SDC-based) with a 10 mg/mL db-ECM concentration.

Creep-recovery test was then performed on db-ECM hydrogels to assess the effect of varying decellularization methods on the viscoelasticity of resulting hydrogels. A stress of 0.1 Pa was applied onto hydrogels while monitoring strain over time, which was followed by a relief of applied stress in a time-dependent manner. To demonstrate the altered viscoelastic properties as a result of different decellularization methods, permanent strain, which indicates the degree of viscoelasticity, was calculated (Figure 5b,e). Interestingly, although hydrogels obtained through methods D (SDC-based) and F (physical-SDC-based) had the same db-ECM concentration and similar stiffness, they exhibited very different stress relaxation. The permanent strain of method D (SDC-based) hydrogels was significantly higher than that of method E (SDS-based) hydrogels (Figure 5e). Method F (physical-SDC-based) hydrogels, despite their higher stiffness, had very similar permanent strain to method D (SDC-based) hydrogels at a constant db-ECM concentration of 10 mg/mL. Method G (Triton X-100-SDC-delipidization-based) hydrogels with higher ligand concentration (20 mg/mL) and increased stiffness compared to method D (SDC-based) (10 mg/mL) hydrogels demonstrated similar permanent strain. On the other hand, although method G20 (Triton X-100-SDC-delipidization-based) (20 mg/mL) hydrogels and method F (physical-SDC-based) (10 mg/mL) hydrogels had comparable stiffness, they revealed significantly different permanent stain values, implicating that viscoelastic properties are altered by the decellularization process. The creep-recovery test was not achieved on sample G10 (Triton X-100-SDC-delipidization-based) (10 mg/mL) since the gel-like structure was destroyed during the creep application, so the measurement for G10 was excluded. These results reveal that the decellularization method highly impacts the distinct mechanical characteristics of reconstituted db-ECM hydrogels and that via using different methods, tunability of ligand density, stiffness, and viscoelasticity could be achieved.

### 2.5 Cellular Growth and Morphology in db-ECM Hydrogels

**Figure 6:**
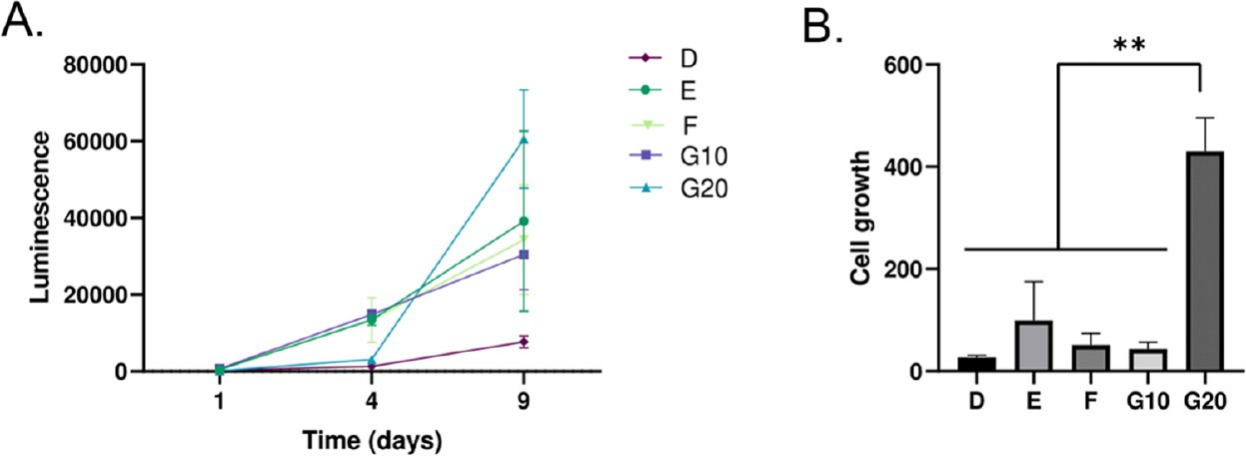
Cellular viability and growth of neuroblastoma cells encapsulated in the db-ECM hydrogels. (a) Growth curve of neuroblastoma cells in db-ECM hydrogels monitored with metabolic activity on days 1, 4, and 9. (b) Comparison of cell growth in terms of metabolic activity between different db-ECM hydrogels on day 9, normalized to day 1.

The cytocompatibility of db-ECM hydrogels was evaluated by encapsulating SH-SY5Y human neuroblastoma cells within hydrogels derived from methods D–G, using designated concentrations, at a cell density of 5 × 10^5^ cells/ml. All hydrogels supported the viability and growth of cells over 9 days of culture (Figure 6a). The highest cell growth was observed in method G hydrogels with 20 mg/mL db-ECM concentration with significantly higher end point metabolic activity (Figure 6b). Method D hydrogels, in contrast, demonstrated the lowest growth rate.

**Figure 7:**
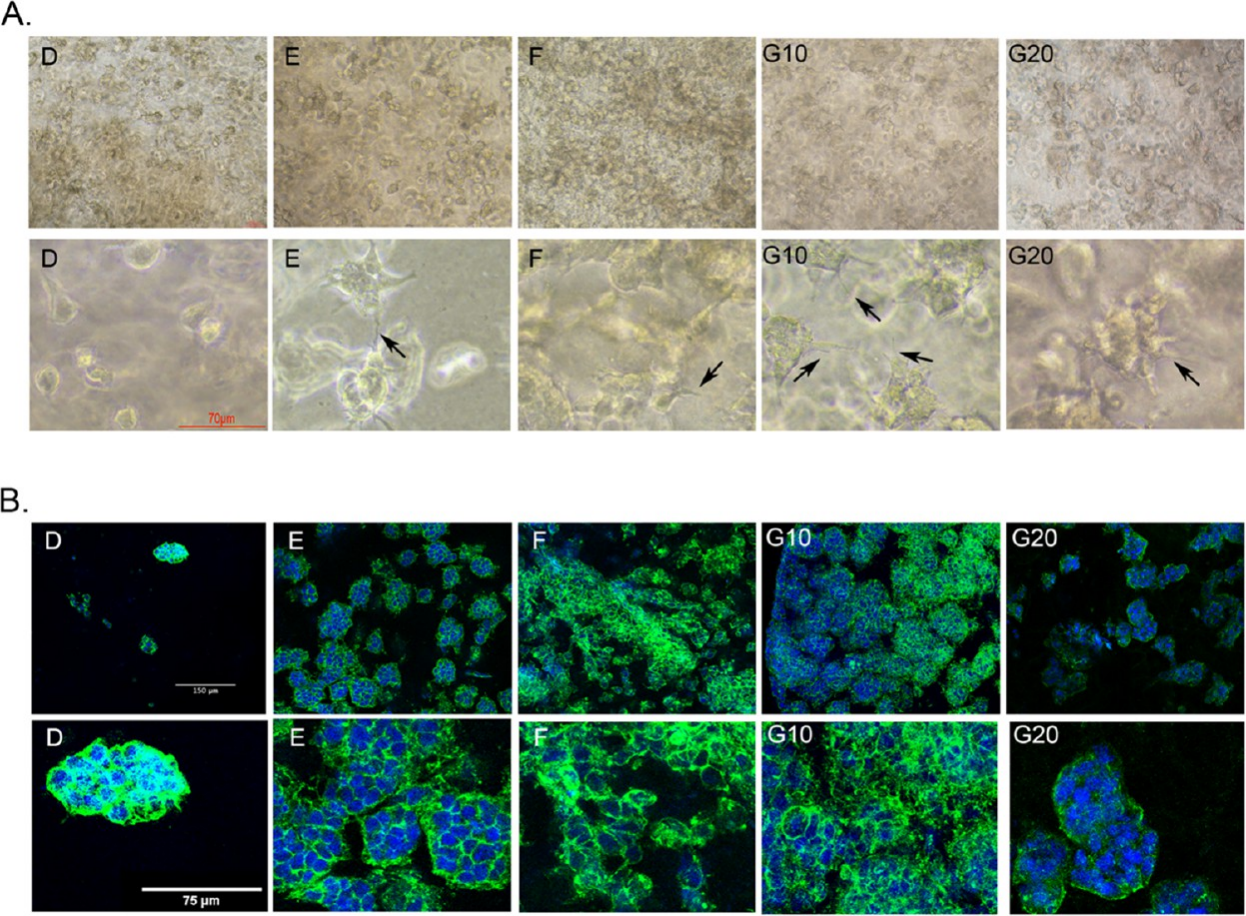
Morphological assessment of neuroblastoma cells encapsulated in db-ECM hydrogels. (a) Brightfield images of 3D neuroblastoma cell-encapsulated hydrogels on day 4. Scale: 100 μm (top panel) and 70 μm (bottom panel). Arrows indicate the cellular protrusions. (b) Immunocytochemical staining images of 3D neuroblastoma cell-encapsulated hydrogels on day 4, green: phalloidin, blue: nuclear staining with DAPI. Scale: 150 μm (top panel) and 75 μm (bottom panel).

Starting from day 4, cells exhibited clump formation and changes in morphological features (Figure 7a,b). In accordance with metabolic activity, the lowest cell density was observed in the method D hydrogels. Phalloidin/DAPI staining was then performed to assess the differences in cellular morphology. Cells in db-ECM hydrogels derived from methods E, F, and G showed distinct cellular protrusion formation (Figure 7a). Furthermore, as opposed to formation of circular cell clumps in hydrogels derived with methods D–G, dispersed and irregular cell growth was observed for method F hydrogels (Figure 7b).

**Figure 8:**
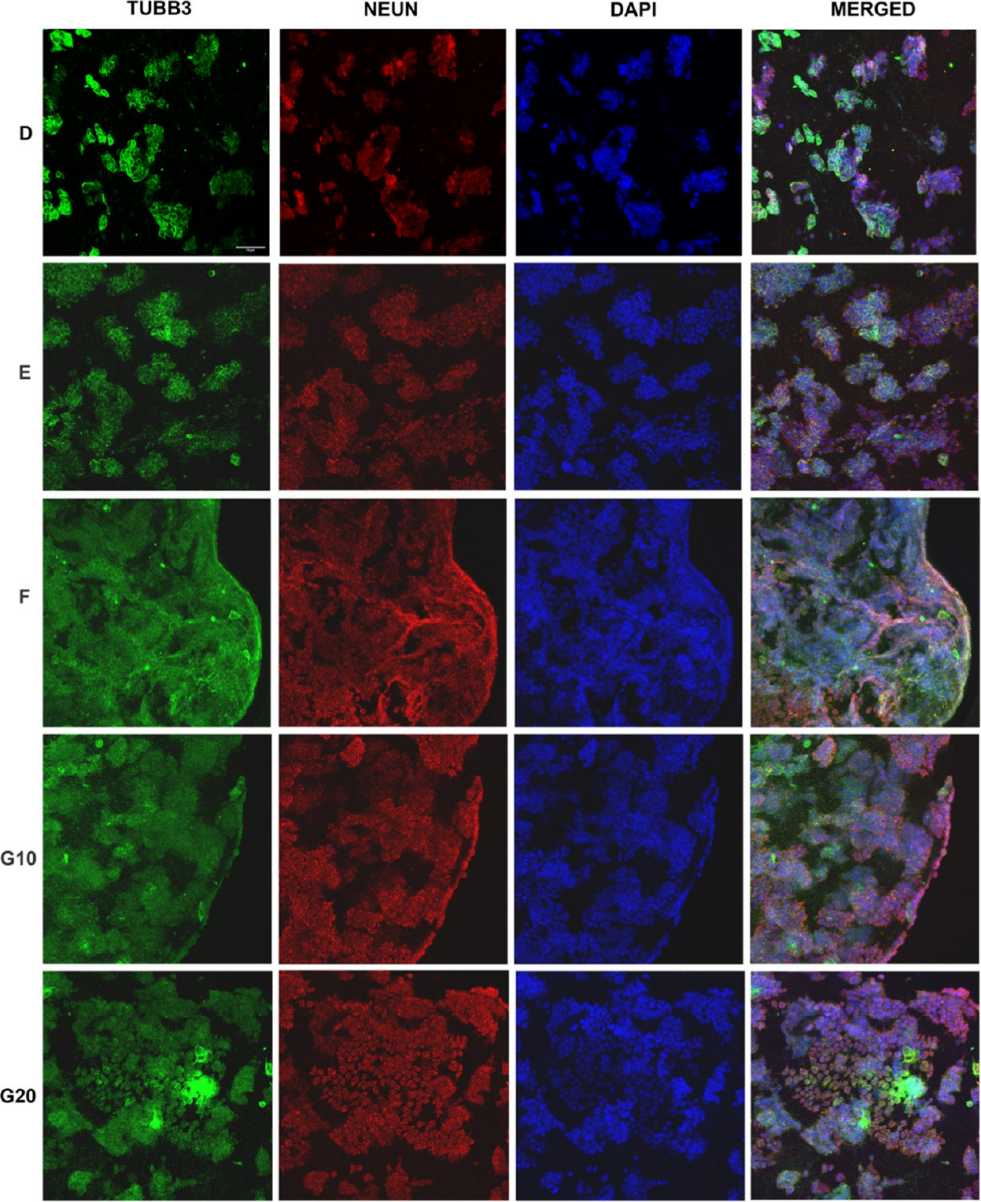
Immunocytochemical staining images of 3D neuroblastoma cell-laden db-ECM hydrogels on day 4. Neuronal markers: β-tubulin III in green, neun in red, nuclear staining (DAPI) in blue (scale bar: 75 μm).

Further immunocytochemical studies were performed in order to assess the expression of neuronal markers in db-ECM hydrogels derived from varying methods. For this purpose, NEUN, a neuronal nuclei marker, and TUBB3 (class III β-tubulin), a mature neuron marker, were investigated in SH-SY5Y cells embedded in db-ECM hydrogels. As shown in Figure 8, cells cultured in hydrogels derived from methods D, E, F, and G were all positive for the selected neuronal markers, indicating that bovine brain-derived db-ECM hydrogels can recapitulate the native brain matrix and support the expression of neuronal markers by neuroblastoma cells.

## 3 Discussion

Generating biochemically and biophysically tunable brain tissue models is an urgent need in neuroscience since there is a progressive increase in the incidence of neurodegenerative diseases. Although conventional 2D *in vitro* models have been in use for long, they fail to recapitulate the complex native extracellular microenvironment and unique mechanical properties of brain tissue. *In vivo* models also have drawbacks, including discrepancies in cellular behavior, divergent genetic expression patterns, differences in cortical progenitor subtypes, and morphological variations.^44^ Therefore, 3D engineered human models offer a desirable choice for studying the molecular physiology of the brain within a microenvironment that represents the key biochemical and mechanical features.^45,46^

Hydrogels, one of the frequently pursued scaffolds in 3D models, resemble native tissues with many physical aspects such as high water capacity, modulated elasticity, and mass transport features.^47^ Decellularized organ-derived hydrogels offer distinct advantages such as preservation of native, tissue-specific ECM cues and providing a more physiologically relevant, “familiar” microenvironment for the cells. On the other hand, although commercial reconstituted basement membrane (rBM) hydrogels such as Matrigel have become gold standard 3D carriers, their tumor-derived origin and undefined composition fail to represent the distinct matrix composition and mechanical integrity of native healthy tissues.^48,49^ Collagen, hyaluronic acid (HA), and fibrin offer a more defined composition, which led to their extensive use in tissue engineering, though they overemphasize cellular signaling with a single ECM moiety instead of providing a fine-tuned complex composition seen in organs. Furthermore, an abundance of HA and collagen in the cellular microenvironment has been implied in tumorigenic behavior in cells.^50−52^ Biopolymers such as agarose and alginate offer a biocompatible and mechanically controllable alternative; however, their inert nature hinders cellular integration and limits their use in the absence of supporting cell instructive cues.^52−54^ Synthetic polymers, on the other hand, allow tunable mechanical features and controlled presentation of selected cellular cues yet lack a wholesome representation of the unique combination of biological signals provided by native ECMs.^50^ Despite the strong advantage of d-ECM hydrogels in recapitulation of organ-specific cues, maintaining the physical integrity and physiologically relevant mechanical properties has been a challenge in the field. Therefore, a better understanding of the decellularization process on the subsequent characteristics of reconstituted hydrogels is crucial for advancing their use.^51^

In this study, we constructed hydrogels from decellularized bovine brain tissue for the first time. We examined seven different decellularization methods, including chemical, physical, biological, and combined approaches, for their effect on the biochemical and biophysical properties of db-ECM hydrogels, as well as the growth and morphology of embedded neuroblastoma cells. We adapted protocols that have been established for porcine and rat brain decellularization with modifications (methods A–E). Additionally, we modified and adapted approaches that have been pursued for different parts of the nervous system (such as sciatic nerve for method G^55^) or entirely different organs such as human pancreas for its high lipid content (method F^10^). Neural tissue engineering applications that highly benefit from nonhuman sources to obtain hydrogels as human donors for brain are very limited. Bovine brain poses as a preferable source with its high anatomical resemblance to the human brain. From a developmental perspective, bovine and humans share similar gestation periods of around 40 weeks, a parameter rendered important for neurodevelopment.^34^ An additional advantage of using bovine brain is its larger size compared to other donors, such as rodents, as decellularization leads to severe size shrinkage. Regarding mechanical characteristics, bovine brain white matter demonstrates higher viscosity than gray matter as in the human brain.^56^

Unquestionably, the brain is the most complex organ in the human body. This complexity relies on its heterogeneous ultrastructure, outstanding mechanical properties with its soft construction and high lipid content, and the presence of gray and white matter. For the present study, the most challenging aspect of brain physiology was the low content of ECM proteins.^15^ For this reason, the selection of the decellularization method is strikingly important, as it should support retention of ECM proteins and enable reconstitution of db-ECM into hydrogel form. To optimize the gelation conditions, several parameters were considered within the scope of this study. Notably, an optimal digestion duration of 24 h was established. We could not obtain any hydrogel formation with methods A, B, and C (Supporting Information Figure 3), and unsuccessful gelation processes were correlated with the loss of collagen upon decellularization (Figure 3c). Collagen retention is a key factor during decellularization, as collagen provides tensile strength and joins the framework of native tissues.^57^ Peracetic acid and freeze–thaw cycles have a disruptive effect on the ECM framework.^57,58^ In line with these, we observed a decrease in collagen in method A, which involved a combinational treatment of SDS with Triton X-100 and peracetic acid. Ionic detergents have been reported to result in collagen disruption; however, we did not encounter such loss in the methods involving SDC and SDS treatment alone.^59^ We observed a remarkable increase in collagen density due to volume shrinkage of the tissue during decellularization methods D, E, F, and G, in line with previous studies that similarly reported increased collagen concentration upon shrinkage.^60,61^ sGAGs are important ECM constituents for brain tissue, which mediate cellular behavior across neural networks.^15,31^ In our study, we observed preservation of sGAG proteins in decellularization method B, whereas a slight reduction was examined in all other options. The reason for preservation of sGAGs might be related to the minimal application of ionic detergents in method B, which entails 1 h of SDC treatment. Lipid removal has emerged as one of the key aspects in decellularization of lipid-rich tissues in previous studies.^10,62^ Lipid content can interfere with hydrogel formation; therefore, efficient delipidization is required for successful construction of hydrogels. We qualitatively assessed the lipid content by Oil Red O staining and showed that long-term (4 days) SDS and SDC treatments (methods E and D) were highly effective in lipid removal (Supporting Information Figure 2). Contrarily, when shorter detergent treatments were combined with delipidation steps, such as homogenization in method F and ethanol/dichloromethane treatment in method G, lipid removal was to a lesser extent. Ethanol/dichloromethane treatment was more effective in the removal of lipids compared to physical homogenization; however, longer detergent treatment was necessary for complete removal.

One of the fundamental checkmarks for successful decellularization is the effective removal of cellular content, commonly validated with DNA content. Successful DNA elimination was achieved except for methods A and B in our study. DeQuach et al. reported that SDC and Triton X-100 did not effectively eliminate cellular content to the same extent as SDS.^31^ Method B lacks DNase and SDS treatment, while method A has a short treatment time with SDS; thus, in line with previous work, SDS might have a potential impact on the effective elimination of cellular material. Conversely, method F, which incorporates a freeze–thaw step in its initial stage, has yielded strong DNA removal despite lacking DNase treatment. Similarly, a study on decellularization of large tendons demonstrated that freeze–thaw cycles, along with detergent treatments, hold greater potential in DNA elimination when compared to detergent treatments alone.^63^ The efficacy of method C in DNA elimination could also be attributed to the application of freeze–thaw cycles before the detergent treatments. Ultimately, the SDC-based method (method D) revealed the most potent DNA removal. Overall, methods that involved extended periods of detergent treatments in combination with DNase or physical disruption exhibited the most effective results.

Although there are numerous studies involving decellularized tissue ECM, only a small portion of these demonstrated the effect of decellularization on the biophysical properties of hydrogels, such as mechanical stability and stiffness. It has been established in the literature that mechanical characteristics have an important impact on cell growth, motility, and tissue homeostasis.^64^ In Kingshott et al.’s work, it was postulated that matrix stiffness has a direct effect on cell behavior as cells continuously remodel the matrix.^65^ In the study by Healy et al., the self-renewal and differentiation capacities of neuronal stem cells were observed in different biochemical and physically well-defined microenvironments. Low stiffness was shown to inhibit differentiation and cell spreading, whereas higher stiffness matrices induced neuronal and glial differentiation.^66^ Here, we show that distinct decellularization methods result in hydrogels with different mechanical properties. In addition, the stiffness of our bovine db-ECM hydrogels (Figure 5c) was in a similar range compared to hydrogels obtained from the brains of other animals.^67,68^ We also demonstrated the tunability of ECM content and, hence, ligand density in method G hydrogels, which correlated with an increase of hydrogel stability and stiffness. Cell proliferation was stimulated in response to an increase in ligand density and stiffness (Figure 6).

Viscoelasticity refers to the ability of tissues and organs to exhibit both viscous and elastic properties when subjected to physical forces. Recent studies state that ECM viscoelasticity is a crucial regulator in cancer cell proliferation and migration.^69^ Clinical studies indicate a correlation between viscoelasticity and brain-related diseases. According to Braun et al.’s study, gender and age have a direct effect on brain viscoelasticity.^70^ Apart from aging, diseases such as multiple sclerosis were also connected to changes in brain viscoelasticity.^30^ For this reason, it is crucial to identify and tune the viscoelastic properties of engineered brain tissues. To the best of our knowledge, this is the first study to characterize the effect of decellularization methods on the viscoelasticity of db-ECM hydrogels. Our creep-response data show that bovine brain-derived hydrogels are stress-relaxing materials and that the method of decellularization has a big impact on the relaxation kinetics of hydrogels. In particular, method F hydrogels demonstrated faster recovery compared to G20, and although they had similar gelation kinetics and storage moduli, their time-dependent viscoelastic properties were distinct. We also note that this difference might explain the variance in the metabolic activity and growth of cells in these mechanically distinct db-ECM gels. Recent studies also suggest that stress-relaxing ECM can induce the expression of proteolytic enzymes in cells, which promotes matrix remodeling that could further explain the differences in cell behavior.^71,72^ Overall, the decellularization method has a large effect on the viscoelastic behavior of reconstituted hydrogels, and characterization of the mechanical properties of db-ECM hydrogels is vital since these aspects have important implications on cell behavior. Our results show that choice of decellularization affected the growth and morphology of neuroblastoma cells. In method F hydrogels, cells demonstrated a more dispersed and singular growth trend as opposed to other hydrogels, which showed circular clump formation, typical of neuroblastoma cells in 3D hydrogels.^73^ The higher stiffness of method F hydrogels when compared to methods D, E, and G10 hydrogels might account for the loss of clump formation, as increased stiffness in the microenvironment has been shown to induce invasive phenotype and decreased circularity in cellular clumps.^74,75^ Although G20 and F hydrogels had similar stiffness, increased ECM ligand density in G20 hydrogels might have acted to compensate for the stiffening.^76^ Furthermore, the permanent strain of method F hydrogels was significantly higher than G20, which could potentially induce a more dispersed growth.^77^

Apart from promising advantages, there are also drawbacks of using d-ECM such as batch-to-batch variability and maintenance of sterility. Our data showed that bovine donors revealed low variability in biochemical content, which renders this species a suitable source for reliable modeling of the brain microenvironment. Regarding sterilization, we pursued antibiotic treatment of tissues after decellularization followed by working under sterile conditions, which enabled us to prevent contamination in cell culture. This way, we aimed to avoid the potential adverse effects of terminal sterilization techniques such as ultraviolet irradiation or ethylene oxide on the composition of the db-ECM.^51,78^

## 4 Conclusions

In neuroscience studies, mimicking the body’s most complex organ, the brain has always been a challenge. 3D engineered tissue models with human-derived cells where the key microenvironmental aspects of native tissues could be recreated have gained much attention. Native tissue decellularization offers maximal recapitulation of organotypic ECM, which can then be constructed into a hydrogel form. In the present study, we evaluated different decellularization methods, including chemical, physical, and biological approaches, which were applied to bovine brain tissue. Our findings indicate that choice of method highly impacts the biochemical content of d-ECM and the mechanical properties of the resulting reconstituted hydrogels. We have characterized the effect of decellularization on the viscoelasticity and stiffness of hydrogels for the first time and shown that varying the decellularization method allows for tunability of distinct mechanical aspects that affect the growth and behavior of neuronal cells. Brain ECM undergoes changes in viscoelasticity and stiffness in neurodegenerative disease progression, such as Alzheimer’s disease. Therefore, our db-ECM hydrogels offer a modular organotypic brain tissue model with potential applications in modeling neurodegenerative diseases.

## 5 Methods

### 5.1 Decellularization Methods

Fresh bovine brains, sourced from calves aged between 10 months and 2 years, were obtained from a local slaughterhouse and transported in a sealed plastic container on ice. After rinsing the brains with 2% Penicillin–Streptomycin (P/S) containing distilled water, the cerebellum was carefully separated, and the cortex of brain tissues was dissected into small pieces (1 × 1 × 1 cm^3^) with a scalpel and scissors. Seven decellularization methods were pursued. Three bovine brains were collected for assessment of biochemical content. All treatments were done under magnetic rotation.

(A) SDS, DNase, Triton X-100, peracetic acid: this protocol was adapted from a study involving decellularization of pig brain cortex with minor modifications.^79^ The brain tissue pieces were rinsed in sterile water solutions, including 2% P/S, 10 times. The brain tissues were then incubated in distilled water for 48 h. The tissue pieces were transferred to a 0.2% SDS solution in phosphate-buffered saline (PBS) for 24 h and afterward washed with PBS for 15 min. Tissues were treated with 40 U/mL DNase in 10 mM magnesium chloride (MgCl_2_) buffer at pH 7.5 for 12 h at room temperature, 0.2%Triton X-100 in PBS for 72 h at 4 °C, PBS wash for 15 min, and 0.1% peracetic acid in 4% ethanol for 2 h at 4 °C. Lastly, the remaining tissues were washed with distilled water without P/S 8 times.
(B) Trypsin/EDTA, Triton X-100, sucrose, SDC, peracetic acid: the protocol established for porcine brain decellularization was applied.^80^ Brain tissue pieces were rinsed in sterile water with 2% P/S at 4 °C for 12 h. The following treatments were done sequentially: 0.05% trypsin/EDTA in PBS at 37 °C for 1 h, 3% Triton X-100 in PBS for 1 h, 1 M sucrose for 15 min, distilled water for 15 min, 4% SDC in distilled water for 1 h, 0.1% peracetic acid in 4% ethanol for 2 h, and PBS wash for 15 min. Lastly, the remaining tissues were washed with distilled water overnight at 4 °C.
(C) Freeze–thaw, Triton X-100, SDC, DNase: treatments for decellularization of rat brain were adapted to decellularize the bovine brain tissue.^81^ Brain tissue pieces were exposed to cyclic freezing at −80 °C for 5 h and thawing at 37 °C completely in PBS 4 times. The tissue pieces were incubated in distilled water containing 1% P/S for 72 h at room temperature. The following treatments were done sequentially: 1% Triton X-100 in PBS for 1 h, wash with distilled water for 30 min, 4% SDC in distilled water for 1 h, wash with distilled water for 30 min, 40 U/mL DNase in 10 mM MgCl_2_ buffer at pH 7.5 for 1 h, and lastly, the remaining tissues were rinsed with distilled water for 3–4 h.
(D) SDC, DNase: Simsa et al.’s protocol for porcine brain decellularization was applied with minor adjustments.^68^ The brain tissue pieces were washed with distilled water for 4–5 h at 37 °C, and the solution was drained with a sieve. Then, 1% SDC solution was added, and the solution was changed daily. After 4 days, the remaining tissues were rinsed with distilled water for 5 h and treated with 40 U/mL DNase in 10 mM MgCl_2_ buffer overnight. Finally, the tissue pieces were washed with distilled water several times over 4–5 h.
(E) SDS, DNase: we followed the protocol for porcine brain published by DeQuach et al., with slight modifications.^31^ The brain tissue pieces were incubated in 0.1% (w/v) SDS in PBS solution with 1% P/S. The solution was changed each day, and incubation lasted for 4 days. Afterward, tissues were treated with 40 U/mL DNase in 10 mM MgCl_2_ buffer at pH 7.5 for 2 h. The thick slurry was separated into falcon tubes and washed with distilled water with sequential centrifugation at 10,000 rpm for 5 min 10 times.
(F) Freeze–thaw, homogenization, SDC**:** we adapted a decellularization protocol developed for human pancreas, a similarly high-lipid-containing tissue as the brain, with adjustments to achieve bovine brain decellularization.^10^ Brain tissues were frozen at −80 °C and thawed at 37 °C. Then, the tissue pieces were washed with PBS for 30 min, washed with distilled water, and homogenized in water until they became a thick slurry. The homogenate was then centrifuged at 4300 rpm for 5 min, and the fat and supernatant layers were discarded. After centrifugation, the pellet was dissolved in 2.5 mM SDC in PBS and incubated for 3 h at room temperature on a rotator. The solution was renewed and incubated for a further 15 h. Then, the remaining tissue was rinsed with water and washed with PBS containing 1% P/S for 72 h, with daily solution changes performed 3 times.
(G) Triton X-100, SDC, delipidation: this methodology was used to decellularize sciatic nerves extracted from pigs, which we adapted to bovine brain decellularization with minor modifications.^55^ The tissue pieces were put in a beaker soaked in distilled water for 6 h. Then, the following treatment steps were followed: 3% Triton X-100 in PBS for 12 h, distilled water containing 1% P/S for three washes, 4% SDC for 24 h, distilled water containing 1% P/S for three washes. Afterward, the remaining tissue was lyophilized until the tissue was completely dried and treated with ethanol/dichloromethane (1:2 v/v) for 24 h. The tissue was washed with sterile distilled water.

Sterilization was achieved by conducting additional wash steps with distilled water containing 1% P/S. Following the decellularization methods, a small sample fragment of decellularized tissue was stored for further histological and biochemical analysis, and the remaining tissue pieces were collected in a falcon tube and stored at −80 °C before lyophilization. Lyophilization was performed until the tissue pieces were completely dried.

### 5.2 Decellularized Brain ECM (db-ECM) Solubilization and Hydrogel Formation

Following the decellularization procedure, the db-ECM samples were lyophilized until completely dry and then cryomilled. Next, 1 mg/mL pepsin was dissolved in 0.1 M hydrochloric acid (HCl), and powdered db-ECM (10, 20, and 40 mg/mL) was added into the pepsin solution to digest for 24 h at room temperature on a magnetic stirrer. Although db-ECM samples were solubilized to a great extent during digestion, in order to remove the possible remaining insolubilized fiber traces and ensure a homogeneous subsequent gelation process, the digest samples were centrifuged, and hydrogels were formed from the supernatant of the samples. After digestion was completed, the digest solution was centrifuged at 13,000 rpm for 10 min, and the solubilized db-ECM in the supernatant was reserved. Solubilized db-ECM was neutralized on ice with cold sodium hydroxide, and the pH was adjusted to physiological pH, 7.4 ± 0.2. The formation of hydrogel was achieved by incubating the neutralized and solubilized db-ECM at 37 °C for 1 h. The neutralized digest was stored at −20 °C and lyophilized. The lyophilized digest was kept at −20 °C until further usage and dissolved in sterile cell culture medium, including 1% P/S and 50 μg/mL Fungin (Invivogen, #ant-fn-2).

### 5.3 Measurement of the DNA Content

The DNA content of brain ECM was quantified using a Quant-iT PicoGreen dsDNA assay kit (Life Technologies Corporation, Carlsbad, CA). Native and decellularized wet tissues were sectioned into 5 mg samples. The samples were digested in 500 μL of papain buffer (pH 6.3), including 100 mM EDTA, 100 mM sodium phosphate, 10 mM l-cysteine HCl, and 10 mg/mL papain at 60 °C for 21 h. The samples were vortexed every 4–5 h. After incubation, the samples were centrifuged, and the supernatants were diluted with 1× TE buffer. 100 μL of diluted samples and dsDNA standards were transferred to a solid black 96-well plate. 100 μL of picogreen solution was added to each well. After incubation for 5 min in the dark at room temperature, fluorescence was measured using a plate reader with an excitation wavelength of 485 nm and an emission wavelength of 520 nm. To confirm the DNA elimination in the decellularized tissues, we performed gel electrophoresis for extracted DNA samples from native and decellularized tissues. For this purpose, 1% agarose gel in tris-borate-EDTA containing ethidium bromide was used. The samples were mixed with gel loading dye 6×, no SDS (NEB, #7025), and, as a reference, GeneRuler 100 bp DNA ladder (Thermo Scientific) was used. The gel was run at 80 V for 60 min and visualized with ChemiDoc XRS+ Imaging System (Bio-Rad).

### 5.4 Quantification of Collagen Content

The insoluble collagen content of native and decellularized brains was analyzed using the Sircol Collagen Assay (Biocolor, U.K.) according to the manufacturer’s instructions. Shortly, 20–30 mg of wet tissue was weighed and digested with fragmentation reagent (supplied by the kit) at 65 °C for 3 h. Afterward, samples were centrifuged at 12,000 rpm for 10 min, and the supernatant was diluted in a new tube. Sircol dye reagent was added and incubated for 30 min at room temperature. Then, centrifugation was done, and the tubes were drained. Ice-cold acid-salt wash reagent was added and centrifuged again. The tubes were drained again, and 1 mL of alkali reagent was added to dissolve the pellets. The absorbance values of the standards and samples at 550 nm were measured using a microplate reader. Absorbance values were normalized to the sample weight.

### 5.5 Quantification of Sulfated Glycosaminoglycan (sGAG) Content

The sulfated glycosaminoglycan (sGAG) content of native and decellularized brains was quantified by using the Blyscan sGAG Assay Kit (Biocolor, U.K.), following the manufacturer’s protocol. Briefly, 20 mg of wet tissue samples was weighed and digested in papain extraction reagent containing 100 μg/mL papain at 65 °C overnight. Blyscan dye reagent was added, and the precipitated sGAG-dye complexes were dissolved with the dissociation reagent. The absorbance values of standards and samples at 656 nm were measured using a microplate reader. Absorbance values were normalized to sample weight.

### 5.6 Brain Histology

Native and decellularized brain samples were fixed with 3.7% formaldehyde solution at 4 °C overnight. Fixed samples were embedded in optimum cutting temperature solution (OCT, Tissue-Tek), frozen, and 10 μm cryosections were cut and mounted on glass slides. For DNA staining with Hoechst, slides were hydrated and stained for 15 min in 1 μg/mL Hoechst solution (Invitrogen) in PBS and visualized by fluorescence microscopy. For Haematoxylin & Eosin staining, slides were hydrated and stained with Mayer’s Haematoxylin for 3 min, followed by a 3 min wash with tap water. Then, slides were immersed in 95% ethanol and stained with Eosin alcoholic solution for 45 s. For collagen staining, Sirius Red (PolySciences) in a saturated aqueous solution of picric acid was used. Slides were stained for 1 h and then rinsed in 0.5% acetic acid solution. Alcian blue staining was performed for sGAG assessment. Slides were hydrated and stained with 1% Alcian Blue in 3% acetic acid solution at pH 2.5 (Sigma) for 30 min, followed by a 2 min wash with tap water. Delipidization was examined by Oil Red O staining. Slides were rinsed with tap water and incubated in 60% isopropanol. Then, slides were incubated in Oil Red O stain (Sigma) for 15 min and washed with distilled water. After staining, all slides were dehydrated, mounted, coverslipped, and visualized by light microscopy.

### 5.7 Mechanical Characterization

Oscillatory rheology was performed for monitoring storage modulus, loss modulus, and permanent strain of db-ECM hydrogels. 250 μL of the neutralized digested hydrogel sample was poured onto the lower plate, which was precooled to 4 °C, and a 20 mm parallel plate was immediately lowered until the hydrogel filled the gap. Then, the lower plate was heated to 37 °C; storage and loss moduli were measured for 30 min with a fixed frequency of 0.5 Hz and 0.1% strain. When the storage modulus of the sample reached an equilibrium state, a creep-recovery test was performed where 0.1 Pa shear stress was applied for 15 min, strain was measured, and then the sample was unloaded, and strain was recorded over time. All measurements were done in triplicate.

### 5.8 Cell Culture

The human neuroblastoma cell line, SH-SY5Y (ATCC, CRL-2266), was cultured in Dulbecco’s modified Eagle’s medium (DMEM) with 4.5 g/L d-glucose (Sigma, RNBK3727) containing 10% heat-inactivated fetal bovine serum (FBS, Biowest, #S181H-500) and 1% P/S. Cells were seeded into a culture flask at a cell density of 2 × 10^4^ cells/cm^2^ and incubated at 37 °C in a humidified atmosphere with 5% CO_2_. The media was changed every other day, and the cells were passaged when they reached 80% confluence.

### 5.9 Cell Encapsulation in db-ECM Hydrogels

Neutralized db-ECM digests were stored at −20 °C until cell encapsulation. The db-ECM digests were thawed on ice, and 10× PBS was added into the digests. The cell suspension was mixed with the db-ECM digests at a concentration of 5 × 10^5^ cells/mL and cast on a 24-well plate. The plate was incubated at 37 °C for 1 h to allow gelation, and afterwards, complete cell culture medium was added onto cell-laden hydrogels carefully. The culture medium was changed every 3 days.

### 5.10 Cell Viability Assay

The cell-laden hydrogels (5 × 10^5^ cells/mL) were prepared using db-ECM digests and SH-SY5Y cells. On days 1, 4, and 9, CellTiter-Glo 3D assay (Promega) was performed. For this purpose, CTG-3D assay reagent with fresh cell culture medium was added to each well at designated time points. After shaking and incubation for 45 min, the cell culture medium was transferred to a black-wall 96-well plate to measure luminescence using a microplate reader. A growth curve was then constructed for the selected db-ECM hydrogels.

### 5.11 Immunostaining

SH-SY5Y cells were encapsulated in db-ECM hydrogels (5 × 10^5^ cells/mL), which were then fixed with 4% paraformaldehyde (Sigma) after 4 days in culture. For membrane permeabilization, 0.1% Triton X-100 in PBS treatment was performed for 1 h at room temperature. Then, blocking was done with 1% bovine serum albumin solution, including 10% goat serum for 2 h. Primary antibodies anti-Neun (1:300, ab177487, Abcam) and anti-β-Tubulin III (2 μg/mL, #801213, Biolegend) were used for immunolabeling. After overnight incubation at 4 °C in constant rotation, samples were incubated with secondary antibodies Goat anti-Mouse IgG (H + L), FITC conjugated (Jackson Immunoresearch, #115–095–003) and Goat anti-Rabbit, Alexa Fluor 594 (Invitrogen, R37117) were treated in the dark. Nuclear counterstaining was done with 4′,6-diamidino-2-phenylindole (DAPI), and imaging was performed with confocal microscopy. Alternatively, after blocking, another sample set was stained with Phalloidin-iFluor 488 Reagent (Abcam, ab176753) and DAPI (Sigma, #MBD0015) for morphological assessment with confocal microscopy.

### 5.12 Statistical Analysis

The stated quantitative data with mean ± standard deviation (SD) values were performed with minimum *n* = 3 replicates. One-way analysis of variance (ANOVA) with Tukey’s post hoc tests was applied to all experimental data using GraphPad Prism 8 software. *P* values of <0.05 were considered statistically significant. Prism’s recommended classification for significance was followed (*p* < 0.0001 = extremely significant (****), 0.0001 < *p* < 0.001 = extremely significant (***), 0.001 < *p* < 0.01 = very significant (**), and 0.01 < *p* < 0.05 = significant (*)).

## Supporting Information Available

Gel electrophoresis of native and decellularized brain tissues; Oil Red O staining of native and decellularized brain tissues, and images of unsuccessful gelation trials with methods A, B, and C (PDF)
