# Supplementary material for: Developing Bovine Brain-Derived Extracellular Matrix Hydrogels: a Screen of Decellularization Methods for Their Impact on Biochemical and Mechanical Properties
Source: ACS Omega. 2023 Sep 29;8(40):36933–47. doi: 10.1021/acsomega.3c04064 (PMC10569007; doi:10.1021/acsomega.3c04064)
Supplement: Supplementary file 1 — ao3c04064_si_001.pdf [file ao3c04064_si_001.pdf]

Supporting Information

Developing bovine brain-derived extracellular matrix hydrogels: a screen of decellularization methods for their impact on biochemical and mechanical properties

Duygu Turan Sorhun[1,2], *Alican Kuşoğlu*[1,2], *Ece Öztürk*[1,2,3,*]

[1] Engineered Cancer and Organ Models Laboratory, Koç University, Istanbul 34450, Turkey

[2] Research Center for Translational Medicine (KUTTAM), Koç University, Istanbul 34450, Turkey

[3] Department of Medical Biology, School of Medicine, Koç University, Istanbul 34450, Turkey

[*]: Corresponding author; e-mail: ozturkece@ku.edu.tr

E-mails of authors: dturan21@ku.edu.tr; akusoglu20@ku.edu.tr

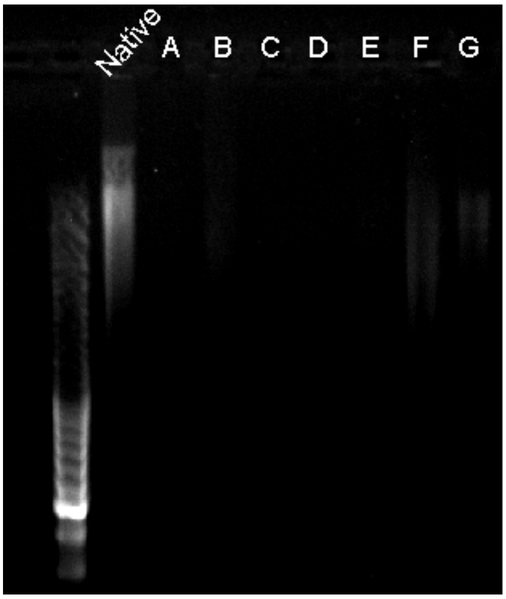

Supplementary Figure 1. Gel electrophoresis of DNA extracted from the native and decellularized brain tissue.

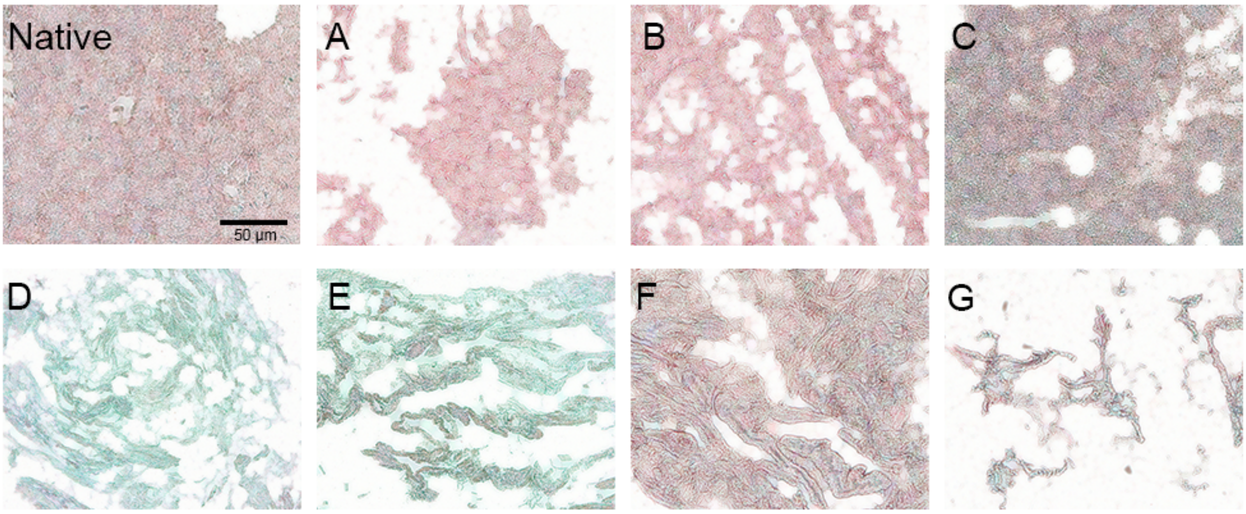

Supplementary Figure 2. Lipid content assessment. Oil red O staining of native and decellularized bovine brain tissues (methods A-G).

Method A_

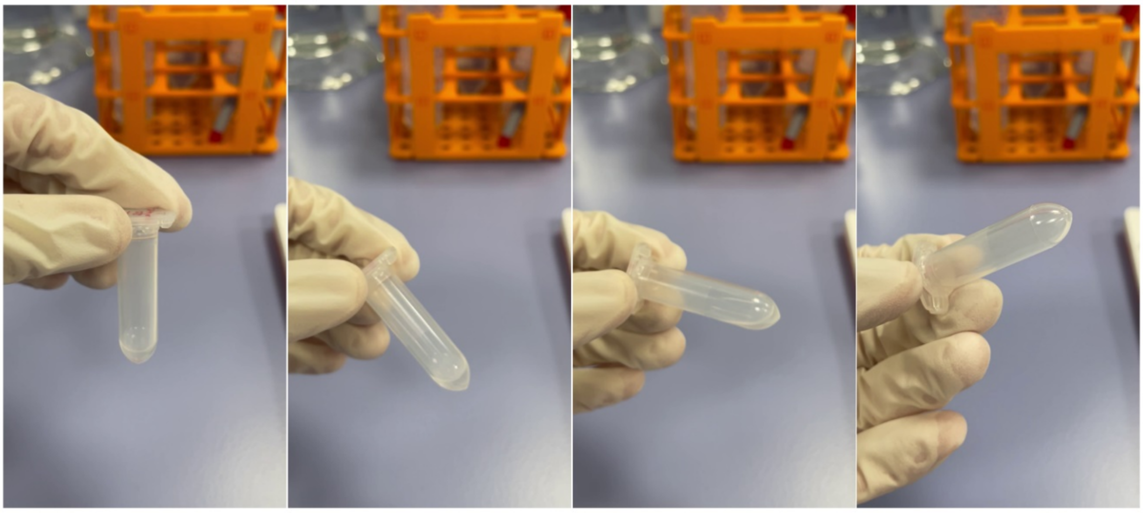

Method B_

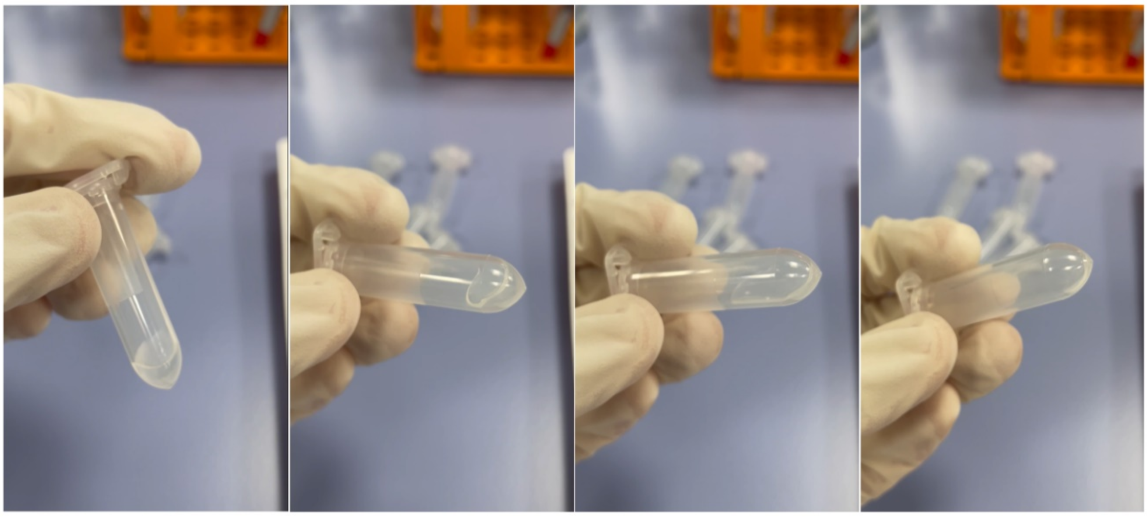

Method C_

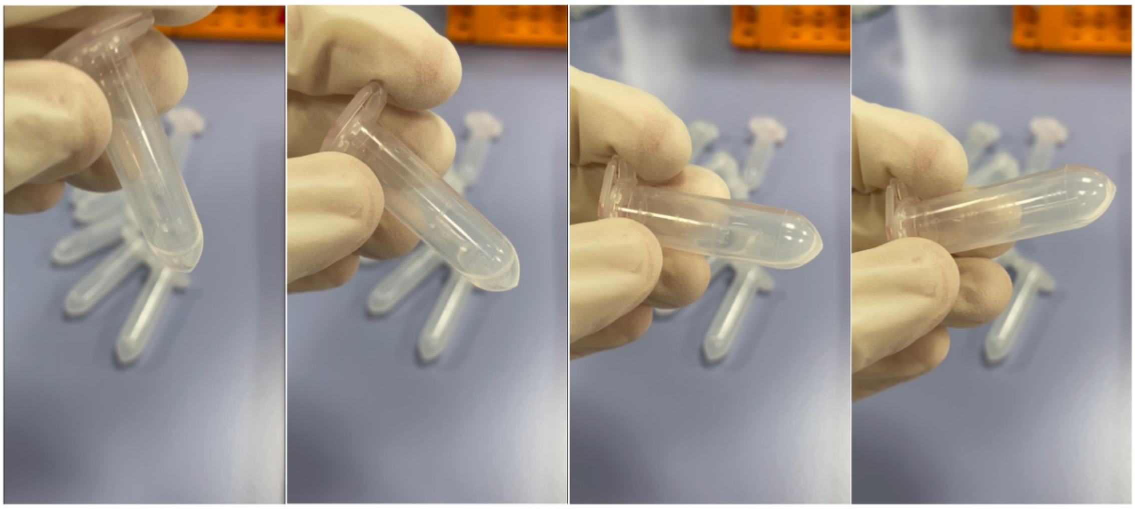

Supplementary Figure 3. Screenshots from experimental videos of unsuccessful gelation methods.
